# Spatial factors affecting young females’ disaster exposure in municipalities facing population decline

**DOI:** 10.1371/journal.pone.0334706

**Published:** 2025-10-17

**Authors:** Jun Sakamoto

**Affiliations:** Faculty of Science and Technology, Kochi University, Kochi, Japan; Thammasat University, THAILAND

## Abstract

Understanding and reducing the risk of natural disasters is crucial for maintaining the economy and society. Since Japan is a disaster-prone country and the most advanced nation in the world regarding aging and population decline, it necessitates a natural disaster risk analysis that considers both challenges. This paper represents the first study to explore the risk of natural disasters, specifically for young females exposed to four types of events: floods, tsunamis, storm surges, and sediment disasters, particularly in municipalities at risk of vanishing. We identified municipalities with a high proportion of young females exposed to these natural disasters and examined them from a geographical perspective. Utilizing a spatial regression model, we statistically analyzed the factors influencing the proportion of the exposed population. The result showed that young females exposed to flooding tend to the areas with a higher proportion of habitable land, inland regions, and locations abundant in rivers and lakes. Conversely, young females affected by tsunamis tend to be in coastal areas with few buildings. Additionally, those exposed to storm surges are often in regions characterized by features such as rice fields and other facilities. In the case of landslides, young females tend to reside in areas where habitable land is scarce, such as coastal regions. Our findings suggest that municipalities with a risk of citywide flooding or storm surge should enhance countermeasures, such as implementing building regulations in high-hazard residential areas. Additionally, it recommends that municipalities with a risk of tsunami or sediment should relocate hazardous housing.

## Introduction

Japan faces an unprecedented challenge of a super-aging and declining population [1,2]. In 2023, 29.1% of the population was 65 years old and over, making it the second-highest proportion globally [3]. In the same year, the number of births was 727,288, while the natural decrease in the domestic population was 831,872. This trend of an aging and declining population is anticipated to continue. Additionally, a persistent migration of young people into Tokyo exacerbates the population decline and aging in rural areas. The Japanese government enacted the Act on the Promotion of Regional Revitalization in 2014 to address this issue. This Act aims to create attractive regions by supporting local government initiatives and facilitating a flow of jobs and people to rural areas. However, despite the passage of ten years since the enaction of the law, the concentration of the population in Tokyo remains unaddressed, leading local governments to compete with one another to attract residents [4].

Natural disasters pose a significant threat to the lives of people in the affected areas and the sustainability of local populations, leading to increased outmigration [5,6]. Research conducted in the United States has shown a positive correlation between the cumulative impact of disasters in the 1990s and changes in the local population and the number of housing units [7]. In the case of Japan, after the Great East Japan Earthquake in 2011, many residents of the impacted areas chose not to return, resulting in a noticeable decline and aging of the population due to a lower birth rate [8]. Likewise, in the aftermath of the Noto Peninsula earthquake in Japan in 2024, the population in the affected six towns and cities of Ishikawa Prefecture decreased by approximately 5,000 within seven months of the disaster. During this period, the number of residents moving out surpassed those moving in, leading to a 4.8-fold increase in outmigration [9]. The finding that areas that were already in decline in terms of population before the disaster were most likely to experience a significant decline in population after the disaster provides an important implication for this study [10]. Therefore, it is desirable for local governments, especially those facing population decline issues, to provide safe places for residents to live regularly to prevent the population decline caused by disasters from accelerating.

## Objective

This research aims to gain insights to help local governments in Japan, which are facing significant population challenges, mitigate future disaster-related difficulties. The case study area is on the 744 municipalities classified as “Municipalities at risk of vanishing” by the Population Strategy Council (Chair: Mimura A.), a group of private sector experts, on April 24, 2024. The Population Strategy Council has defined the “young female population” as an important indicator of reproductive capacity within the population. This indicator comes from the fact that 95% of the total fertility rate, 1.41 in 2012, was attributed to females aged 20–39. Therefore, a continued decline in the young female population could lead to a decrease in reproductive capacity, making it challenging to stop the overall decline in population [11]. The council has classified the municipalities expected to see a decline of 50% or more in their young female population from 2020 to 2050 as “municipalities at risk of vanishing. “

We analyze population forecasts for 2050, disaster hazard data, and spatial information for these municipalities. The first is to identify municipalities with a high proportion of young females at greater risk during disasters and examine the geographical characteristics of the top-ranking municipalities. Then, classifying the factors influencing the high proportion of young females at risk using land use and social, economic, and infrastructure development data.

The municipalities identified as being at risk of vanishing are expected to see a significant decline in their young female population. This study is crucial for exploring measures that local governments should implement to prevent migration to other areas after disasters.

The scope of natural disaster risks in this study comes from hazard maps. In Japan, the government defines the population living in high-disaster areas, such as flood-prone zones, on hazard maps as the population within disaster risk areas [12]. They publish the analysis results of future disaster risk based on the definition. It serves as a resource for local governments, businesses, residents, and others to consider appropriate land use from a medium- to long-term perspective in relation to disaster risk. This study analyzes the disaster exposure of young females using the same concept as the “ population living in high-disaster areas.” Hence, the study has limitations, as it does not consider the structural resilience of buildings and local disaster-related laws established by municipalities.

## Literature review and development of the hypothesis

With the increasing availability of spatial data, research on disaster assessment using wide-area risk maps is on the rise. For instance, a study on disaster vulnerability in India identified districts particularly susceptible to disaster damage [13]. Another study focused on Japan visualized the population exposed to disasters in more detail than just municipalities [14]. Additionally, a study in northwestern Iran examined the temporal and spatial changes in vulnerability to floods over 30 years starting from the early 21st century [15].

Changes in land use can increase disaster risk [16–18]. Disaster risk differs depending on hazard characteristics, and areas with high sediment-related disaster risk are places where climatic, topographical, and hydrological factors overlap with areas of high population density, road development, and urbanization [19,20]. Areas with high flood risk are places with forests and urban areas in low-lying areas [21]. Appropriate land use planning by local governments reduces serious human and material damage caused by natural disasters [22]. Strengthening land use regulations in high-risk areas can reduce the likelihood of people settling in high-risk areas [23].

A significant relationship exists between natural disasters and their impact on regional communities, economies, and social infrastructure. For instance, residents of those involved in agriculture are particularly vulnerable to flood damage [24], while fishermen’s homes are at risk from tsunamis and storm surges [25]. In disaster-prone areas, it is advisable to encourage young people or new residents to relocate to safer locations. At the same time, it is realistic to build physical measurements to protect the elderly and those with limited mobility [26]. Additionally, since disasters can adversely affect municipal budgets, implementing land use regulations as a preventive measure is recommended [27].

This study verifies the following hypotheses regarding various disasters, drawing on findings from other countries and analysis results specific to Japan’s topography and environment.

**Hypothesis 1:** Municipalities where buildings are widely spread across the city and where rivers are prevalent have a higher proportion of the population exposed to flooding. Rivers in Japan typically have steep slopes, which can lead to rapid flooding during heavy rainfall. For instance, a case study of low-lying areas in Saga Prefecture, Japan, revealed that urbanized areas with low-rise buildings are at a high risk of flooding [28].

**Hypothesis 2:** Municipalities with small coastal settlements in suburban areas have a higher percentage of the population vulnerable to tsunamis. During the Great East Japan Earthquake, regions with small coastal communities suffered significant damage [29,30]. Furthermore, the anticipated Nankai Trough earthquake is expected to necessitate evacuation measures for coastal residents [31].

**Hypothesis 3:** Municipalities characterized by low-lying areas or ports face a greater risk of storm surges for their populations. Areas at sea level or in bay inlets are particularly susceptible to damage from storm surges. A storm surge inundation model that considers global warming predicts extensive flooding in Kyushu and its three major ports, Japan [32].

**Hypothesis 4:** Municipalities with newly developed residential areas in mountainous regions have a higher proportion of the population at risk of landslides. During Japan’s high-growth economic period, many new towns were built in mountainous areas near suburban regions. In the heavy rains that struck western Japan in 2018, landslides caused significant damage in these newly developed residential areas [33].

**Hypothesis 5:** Municipal development in social, economic, and infrastructure areas is highly vulnerable to disasters. Factors such as an aging population, sewage coverage, and financial conditions are commonly used as indicators of disaster risk [34,35].

## Methodology

### Study design

This study evaluates the hazard exposure of young females by following three stages. The first is to select “municipalities at risk of vanishing” as the case study area for our research. Then, we define the population of young females at high risk in 2050 as the “percentage of young females at high risk” of the total population and aggregate it by the municipality and hazard level. This calculation method is simple and is the value obtained by dividing the young female population in the only high hazard meshes by the young female population in all meshes. The second stage is to create frequency tables for the “percentage of young females at high risk” associated with each hazard. Then, we identify the high-ranked municipalities based on their geographical features and investigate the reason for high hazards and other relevant factors. The third stage analyzes the factors influencing the “percentage of young females at high hazard risk.” This analysis used a spatial autoregressive model, considering both land use and social, economic, and infrastructure development conditions in the municipalities as a potential independent variable. Finally, we discuss countermeasures that municipalities with a large population of young females living in high-hazard areas should consider in the future based on the trends identified from our model.

### Study area

This study analyzes 744 municipalities classified as “Municipalities at risk of vanishing,” as defined by the Population Strategy Council in 2024. The definitions of the specific term and ongoing discussions related to this issue in Japan are as follows.

On May 8, 2014, the Japan Policy Council (Chair: Masuda H.), a private research organization, proposed strategies to address the declining birthrate and revitalize rural areas [36,37]. The proposal stated that if the current demographic trends in rural areas continue, there is a significant risk that regions experiencing a high outflow of young females (aged 20–39) and rapid population decline will ultimately disappear, regardless of any increases in the birth rate. Furthermore, the “Projected Population of Japan by Region (estimates for March 2013)” published by the National Institute of Population and Social Security Research (IPSS) predicts that if regional migration rates are assumed to stabilize, municipalities where the population of young females will decrease by more than 50% by 2040 could total 896 (excluding municipalities in Fukushima Prefecture due to the impacts of the Great East Japan Earthquake) [38]. The Japan Policy Council designated these municipalities as “Municipalities at risk of vanishing” (“Shometsu Kanosei Toshi” in Japanese).

On April 24, 2024, the Population Strategy Council (Chair: Mimura A.), composed of private sector experts, examined this situation from the perspectives of “natural population decline” and “social population decline” (Population Strategy Council, 2024). Based on IPSS’s estimates, the council defined the municipalities expected to see a decrease of more than 50% in their young female populations over the 30 years from 2020 to 2050 as “municipalities at risk of vanishing” (“shometsu kanosei jichitai” in Japanese), and disclose the leading to the creation of a list of these municipalities. The updated count of municipalities at risk of vanishing is 744 (or 711 if those in Fukushima Prefecture are excluded). It reflects the removal of 239 municipalities from the previous list and the addition of 99 municipalities (66 if excluding Fukushima Prefecture) [39].

**Fig 1:**
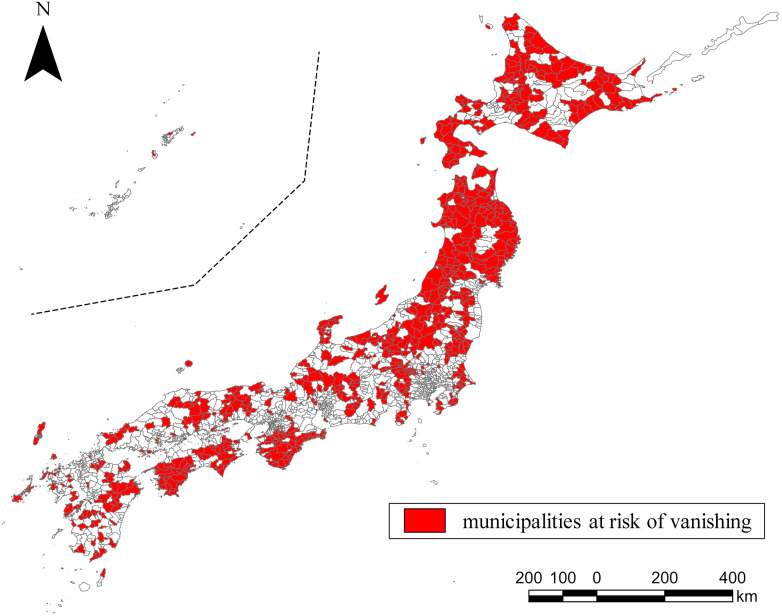
Mapping on municipalities at risk of vanishing. The data of the map is processed from “National Land Numerical Information (Administrative Area Data: Open Data (CC_BY_4.0))” by the Ministry of Land, Infrastructure, Transport and Tourism (https://nlftp.mlit.go.jp/ksj/gml/datalist/KsjTmplt-N03-2024.html). The map was created based on the 744 municipalities designated as “Municipalities at risk of vanishing” by the Population Strategy Council on April 24, 2024 [39].

Fig 1 displays a map of municipalities at risk of vanishing defined in 2024 (n = 744), while Table 1 provides survey results categorized by prefecture. The data indicates that the highest proportions of municipalities at risk of vanishing are in the Tohoku region, particularly in Akita, Aomori, Yamagata, and Iwate. Hokkaido has the highest number of such municipalities, while Okinawa Prefecture has none. Table 2 shows the results of comparing the number of municipalities by population size between municipalities that are at risk of vanishing and other municipalities. The table shows that 45% of municipalities at risk of vanishing have a population of 10,000 or less and that these municipalities are smaller than other categorized municipalities (19%).

**Table 1:** Number of municipalities at risk of vanishing by prefecture. The table was created based on the 744 municipalities designated as “Municipalities at risk of vanishing” by the Population Strategy Council on April 24, 2024 [39].

Rank	Prefecture	Percentage of municipalities at risk of vanishing	Num. of municipalities at risk of vanishing	Other municipalities
1	Akita	96%	24	1
2	Aomori	88%	35	5
3	Yamagata	80%	28	7
4	Iwate	79%	26	7
5	Wakayama	77%	23	7
6	Kochi	74%	25	9
7	Fukushima	70%	33	14
8	Tokushima	67%	16	8
9	Hokkaido	65%	117	62
10	Niigata	60%	18	12
11	Ehime	60%	12	8
12	Gunma	57%	20	15
13	Nara	56%	22	17
14	Oita	56%	10	8
15	Miyagi	54%	19	16
16	Nagasaki	52%	11	10
17	Ishikawa	47%	9	10
18	Fukui	47%	8	9
19	Tottori	42%	8	11
20	Yamaguchi	42%	8	11
21	Mie	41%	12	17
22	Chiba	41%	22	32
23	Yamanashi	41%	11	16
24	Kumamoto	40%	18	27
25	Ibaraki	39%	17	27
26	Gifu	38%	16	26
27	Okayama	37%	10	17
28	Kagoshima	35%	15	28
29	Kyoto	35%	9	17
30	Miyazaki	35%	9	17
31	Nagano	34%	26	51
32	Toyama	33%	5	10
33	Tochigi	32%	8	17
34	Hyogo	32%	13	28
35	Osaka	28%	12	31
36	Hiroshima	26%	6	17
37	Shizuoka	26%	9	26
38	Saitama	25%	16	47
39	Saga	25%	5	15
40	Kagawa	24%	4	13
41	Shimane	21%	4	15
42	Kanagawa	18%	6	27
43	Fukuoka	13%	8	52
44	Aichi	13%	7	47
45	Shiga	11%	2	17
46	Tokyo	3%	2	60
47	Okinawa	0%	0	41

**Table 2:** Comparison of population size between municipalities at risk of vanishing and other municipalities.

Population size	Municipalities at risk of vanishing		Other Municipalities	
300,001–	0	(0%)	83	(8%)
200,001–300,000	3	(0%)	43	(4%)
150,001–200,000	3	(0%)	45	(5%)
100,001–150,000	11	(1%)	91	(9%)
75,001–100,000	8	(1%)	76	(8%)
50,001–75,000	37	(5%)	122	(12%)
30,001–50,000	85	(11%)	149	(15%)
20,001–30,000	85	(11%)	80	(8%)
10,001–20,000	178	(24%)	107	(11%)
–10,000	334	(45%)	189	(19%)
Number of Municipalities	744		985	

### Data

The primary data utilized in this study are 500m mesh population data, 1 km mesh land use data, habitable area data, administrative boundary data from the National Land Numerical Information, and hazard maps provided by the Geospatial Information Authority of Japan. The following sections explain each of these data sources in detail.

The 500m mesh population data is a government estimate in 2018, organized into 500m mesh units [40]. This dataset features the GridCode4 mesh code (a 9-digit integer), the administrative district code (a 5-digit number identifying cities, towns, and villages), the population figures derived from the 2015 national census, and projected population estimates for the years 2020–2050, broken down by 5-year age groups and gender. This study extracts data corresponding to 744 municipalities based on the administrative district code, focusing specifically on the population of young females in 2020 and 2050. The definition of “Young females” is 20–39 years old, followed by the Japan Policy Council. The objective of utilizing 500m mesh units is to analyze each municipality at this granular level. Since the data published by the Japan Policy Council is aggregation data at the municipality level, it is impossible to compile statistics on the percentage of the young female population exposed to disaster risks, which is the aim of this research. Using mesh data aggregation allows for the above-detailed analysis.

**Fig 2:**
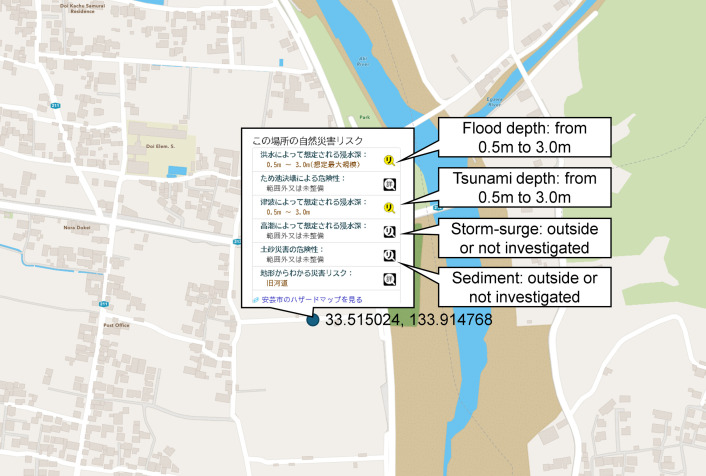
Example of hazard information corresponding to point data. The source file for the map is NC OneMap, and written consent is not required to use this geospatial data. https://www.nconemap.gov/pages/terms. This Fig was created from the hazard map portal site [41].

The land use data provides information on the area of each 1 km mesh, including features such as rivers, forests, agricultural land, building land, lakes, and more, as recorded in 2021. The administrative boundary data outlines municipalities as of 2024. The hazard map portal site allows users to view hazard information for a specific location in a pop-up window by clicking on that location in their browser. For example, Fig 2 shows various hazard information: the probable maximum flood depth ranges from 0.5m to 3.0m, while the tsunami flood depth is between 0.5m and 3.0m. However, the depth of storm surges and the risk of sediment are either outside or not investigated.

The definition of habitable area is subtracting the areas of forests, major lakes, and marshes from the total area. This study uses the habitable area data aggregated by the municipality [42].

### Data processing procedure

**Fig 3:**
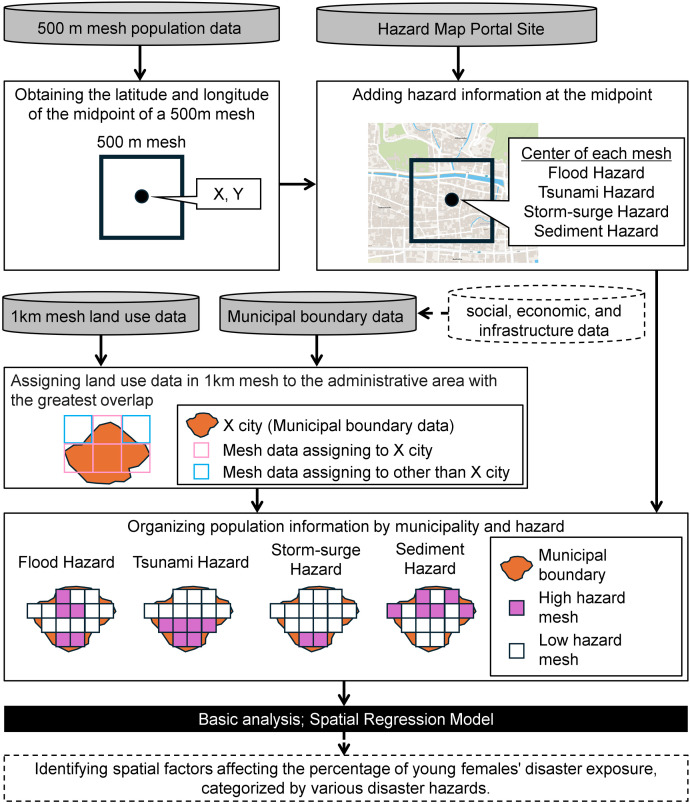
Data processing flow. The source file for the map is NC OneMap, and written consent is not required to use this geospatial data. https://www.nconemap.gov/pages/terms. Spatial data were organized using ArcGIS Pro version 3.4.3, and spatial regression models were performed using Stata BE version 17.0.

Fig 3 illustrates the data organization procedure. The first is to create point data for the midpoints from the vector layer of the 500m mesh population data, yielding coordinates (latitude and longitude) for each point. This process aims to identify a representative location for each 500m mesh, accomplished using the GIS geocoding function. As there are 471,024 500m mesh population data points, the same number of point data points are also generated. We obtain coordinate information through the GIS geocoding function by utilizing this point data.

The second is to combine hazard information associated with the coordinates of each point data using the hazard map portal site [41]. The portal allows users to access “hazard information for specific locations” through a URL incorporating coordinate information [43]. For instance, the coordinates for the point data corresponding to mesh number “503327133” are latitude = 33.51458 and longitude = 133.91563. Thus, we retrieved hazard information by specifying locations as many times as there are point data (n = 471,024).

The third is to align the 1 km mesh land use data with the municipalities in the administrative boundary data. The 1 km mesh land use data that overlaps most significantly with each municipality is combined. Finally, we integrate the administrative boundary and hazard information with the 500m mesh population data. To organize the data by municipality, we utilize the “administrative district code” included in the 500m mesh attribute data in advance. With the data organized through this process, we will analyze the relationship between the young female population and disaster risk/land use in municipalities at risk of vanishing.

**Fig 4:**
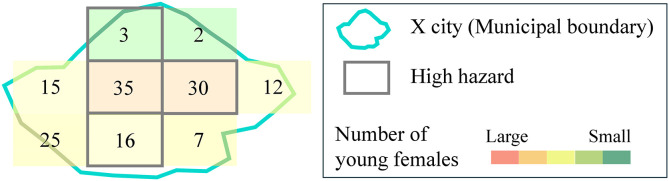
Calculation method of “Percentage of Young Females at High Hazard. ”.

The primary indicator for the following analysis is the “Percentage of Young Females at High Hazard.” To explain how this percentage is calculated, let’s use an example. In X city, as shown in Fig 4, there are a total of 9 meshes, and the population of young females in each mesh in the year 2050 is represented by the number of individuals within that mesh. Among these, 4 meshes are classified as being in a high-hazard area, which includes a total population of 84 young females (3 + 35 + 30 + 16). In contrast, the total population across all 9 meshes is 145 individuals. Thus, the “Percentage of Young Females at High Hazard” is calculated as 57.9%, which is derived from the formula 84/145.

### Data confirmation

Since this study analyzes the young female population in municipalities at risk of vanishing using 500-meter mesh population data, the population figures published by the Population Strategy Council may differ from the calculations made at the municipal level. Therefore, we confirmed any discrepancies between the two datasets to verify the validity of the data used in this study.

**Fig 5:**
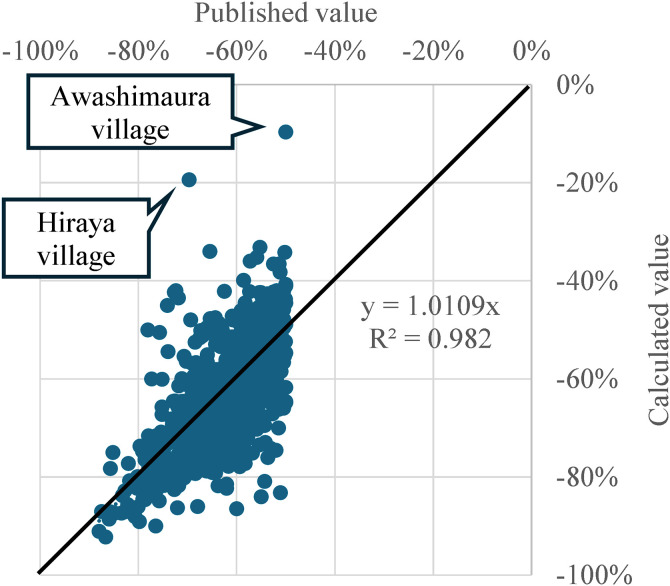
Comparison of the rate of change in young females from 2020 to 2050 between published value and calculated value.

Fig 5 illustrates the rate of change in the young female population (the population ratio in 2050 to that in 2020). The horizontal axis represents the data published by the Population Strategy Council, while the vertical axis shows the calculated values for the 500-meter mesh units. The figure indicates a high R^2^ value of 0.982. However, two municipalities—Awajimaura Village and Hiraya Village—are significant outliers. These municipalities have populations of fewer than 400 (in 2020), and this small sample size influences the calculation of the rate of change for young females.

**Fig 6:**
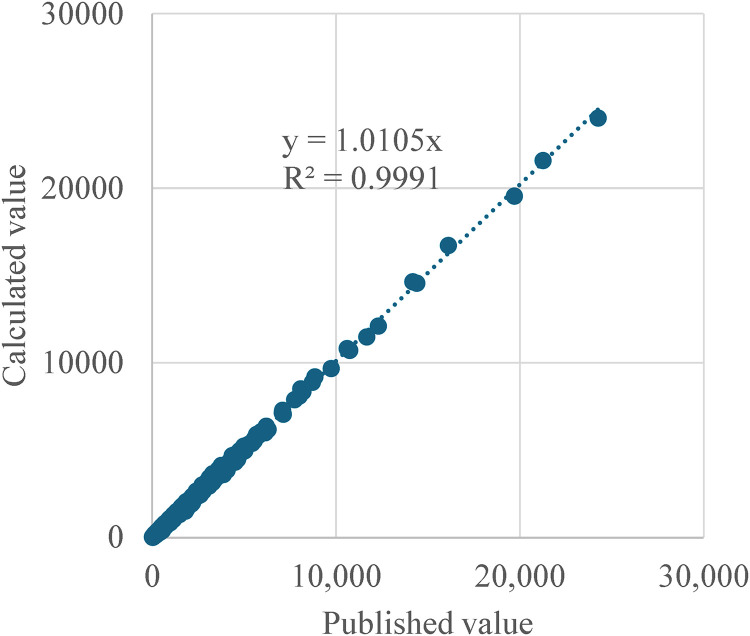
Comparison of the number of young females in 2020 between published value and calculated value.

**Fig 7:**
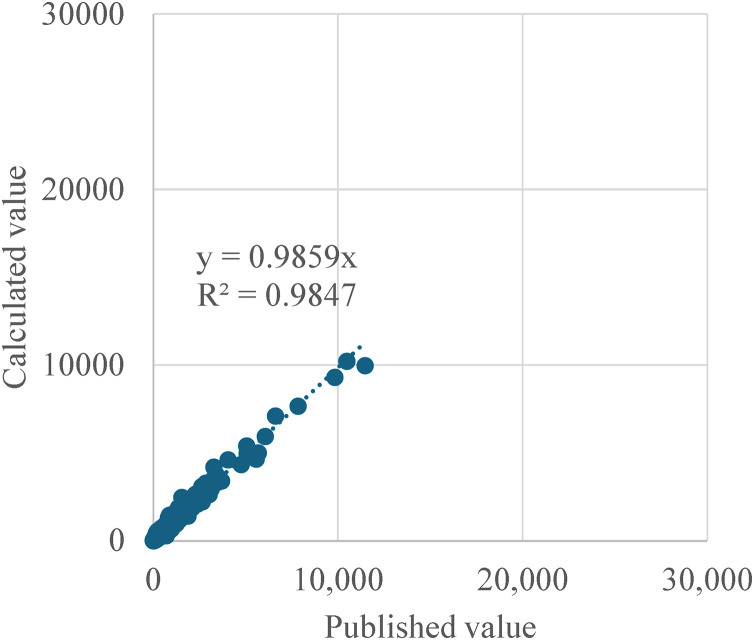
Comparison of the number of young females in 2050 between published value and calculated value.

Fig 6 displays the relationship between the calculated female population and the published female population in 2020, and Fig 7 illustrates the relationship between the calculated and  published female population in 2050. The R^2^ values for both datasets are also high, at 0.982 and 0.9991, respectively. Therefore, we concluded that the calculation methods used in this study align closely with the published figures, indicating no significant discrepancies.

This study develops a spatial regression model utilizing 500-meter mesh population data alongside 1-kilometer mesh land use data. However, due to the differing scales of these two datasets, there is a potential for bias. Since our analysis focuses on the municipality unit, we compare the municipality areas derived from both datasets to assess the extent of any bias.

**Fig 8:**
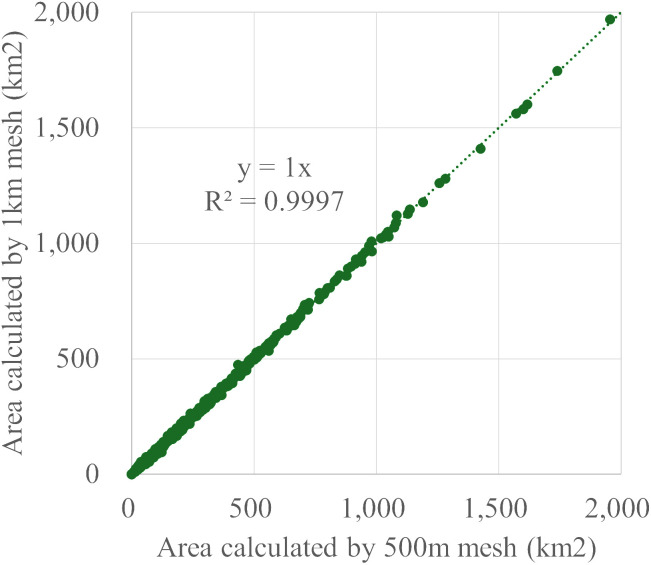
Comparison of municipal area calculated using 500m and 1 km meshes.

Fig 8 illustrates the results of this comparison between municipality areas calculated from the 500-meter and 1-kilometer meshes. It is important to note that this comparison only includes cases where four 500-meter meshes fall within a single 1-kilometer mesh. As depicted in the figure, the correlation between the two mesh sizes is exceptionally high, suggesting that any bias caused by the differences in mesh size is minimal.

### Data conversion

The hazard map portal allows users to view the danger level of a specific location by inputting coordinates. Although the data is available in a unified format across Japan, note that the legend for the assumed danger levels and the scale of potential disasters varies by local government. Therefore, this study focused our analysis exclusively on high-hazard areas after converting the data obtained from the hazard map portal, as detailed in Table 3.

**Table 3:** Conversion of disaster scale. We created the original scale column in the table based on the hazard map portal site [41].

Hazard	Original scale	Modified scale
Flood	Outside Hazard or no data	Low hazard
	Less than 0.3 m	
	Less than 0.5 m	
	More than 0.5 m and less than 1 m	
	More than 0.5 m and less than 3 m	
	More than 1 m and less than 2 m	
	More than 2 m and less than 3 m	High hazard
	More than 3 m and less than 5 m	
	More than 5 m and less than 10 m	
	More than 10 m and less than 20 m	
	More than 20 m	
Tsunami	Outside Hazard or no data	Low hazard
	Less than 0.3 m	
	Less than 0.5 m	
	More than 0.5 m and less than 1 m	
	More than 0.5 m and less than 3 m	
	More than 3 m and less than 5 m	High hazard
	More than 5 m and less than 10 m	
	More than 10 m and less than 20 m	
Storm-surge	Outside Hazard or no data	Low hazard
	Less than 0.3 m	
	Less than 0.5 m	
	More than 0.5 m and less than 1 m	
	More than 0.5 m and less than 3 m	
	More than 3 m and less than 5 m	High hazard
	More than 5 m and less than 10 m	
Sediment	Outside Hazard or no data	Low hazard
	Steep slope hazard area	
	Avalanche hazard area	
	Landslide danger area	
	Landslide hazard area	
	Sediment danger area	
	Sediment disaster special hazard area	High hazard
	Steep-slope landslide special hazard area	

This study focuses on areas with high hazard because it examines the outflow of the population resulting from extensive damage to homes caused by disasters. We have set the threshold for flooding, tsunamis, and storm surges at depths of 2 meters or more for the following reasons. For flooding, research indicates that the first floor of a wooden house is inundated at depths of 2 meters or more [44]. For tsunamis, survey data show that houses are likely to be destroyed at depths of 2 meters or more [45]. Additionally, the Sediment Disaster Special Hazard Area and the Steep-Slope Landslide Special Hazard Area in Japan are locations where strict building regulations have been established to mitigate risks associated with sediment disasters [46].

### Spatial regression model

Spatial autocorrelation refers to the potential interdependence of observation data for various variables within the same distribution area. [47] noted that “all objects on the surface of the earth are related to each other, but the closer the objects are to each other, the stronger the relationship is compared to objects that are further away.” By applying a spatial economic model, it becomes possible to account for the spatial dependence of the data. Since the disaster hazards examined in this research are geographical phenomena with spatial dependence, it is appropriate to consider these spatial relationships in the analysis.

The first is identifying the spatial relationships between observation units to calculate spatial autocorrelation. It means defining which observation units are adjacent. For this purpose, we employ a dimensional neighbor’s weights matrix, denoted as W. Using spectral normalization, we create a matrix that assigns adjacent municipalities the same positive weight while all other municipalities assign a 0.

Since there are various ways to develop a model with spatial dependence, this study adopts three spatial regression methods: the Spatial Autoregressive Model (SAR), the Spatial Error Model (SEM), and the Spatial Durbin Error Model (SDEM). Before developing these models, we utilized Moran’s I statistic to evaluate whether the ordinary least squares (OLS) regression model exhibited autocorrelation. Moran’s I statistic is the most commonly used measure of spatial autocorrelation, which assesses how variables are either concentrated or dispersed in space [48]. The test hypothesizes that the model’s residuals are independent and identically distributed. If the test results are statistically significant, they reject the hypothesis and suggest that developing the Ordinary Least Squares (OLS) model without considering spatial error dependence will lead to biased estimates of the covariates in the model [49,50].

The first model, SAR, restricts spatial autocorrelation to neighboring districts’ outcomes or the dependent variable’s spatial lag. It means that the percentage of young females at high hazard in a specific municipality is influenced by the percentage of young females at high hazard in adjacent municipalities. This relationship is in the following equation.


y=α+ρWy+Xβ+ε (1)

Where y is an N×1 vector of the dependent variable, W is the dimensional neighbor’s weights matrix, X is an N×K matrix of k={1,2,…K} covariates, ε is an N×1 vector of normally distributed disturbances, β is a K×1 vector of parameter estimates, ρ is the autoregressive scalar parameter, and α is the constant parameter.

The second model, SEM, assumes that the percentage of young females at high hazard depends on observed land use and that the error term is spatially correlated. It considers spatial effects that the model does not explain and may explain the observed spatial autocorrelation in the residuals [51]. It explicitly models the spatial autocorrelation between disturbances expressed by a scalar parameter, as shown in the following equation.


y=α+Xβ+u

u=λWu+ε (2)

The third model, SDEM, is an extension of the spatial lag model that limits spatial autocorrelation to the results and to all spatial lags of the predictors and the error term [52]. This model assumes that the percentage of young females at high hazard in a specific municipality is influenced by the percentage of young females at high hazard in neighboring municipalities and other spatial effects that are not accounted for in the model. It represents a vector of spatial spillover effect parameters θ, illustrated with the following equation.


y=α+Xβ+WXθ+u

u=λWu+ε (3)

The software used for the analysis was StataBE17.0, and the commands used referred to the explanations in the [53,54].

Table 4 shows the candidate predictor variables used to predict the dependent variable: the percentage of young females at high hazard. The “Data” column on the right side of the table shows the website from which each item comes. All items are freely available for anyone to download. We utilized data on inhabitable areas and 12 different types of land use. In the social, economic, and infrastructure development variables, we chose the variables by referring to the research [58,59], which examines regional population movements and economic fluctuations. These variables include population size, aging rate, industry-specific labor force ratio, sewage treatment coverage ratio, number of public facilities, and the ratio of inhabitable area to the total municipal area.

**Table 4:** List of independent variables.

Item		Definition	Data
Population		2020 Census	[55]
Ratio of elderly population		Percentage of population aged 65 or older (2020 Census)	[55]
Ratio of workers	primary sector	2020 Census	[56]
	secondary sector		
	tertiary sector		
Financial strength index		An index, calculated by dividing standard fiscal revenue by standard fiscal needs, indicates local governments’ fiscal strength.	[56]
Ratio of sewage treatment coverage		2024 edition	[57]
Number of public facilities		2022 edition	[55]
Ratio of inhabitable area to total municipal area		Calculated by subtracting the area of the main lakes and marshes from the area of the field	[56]
Ratio of the area of each land use to the area of the municipality	Rice fields	Wet rice fields, dry rice fields, rice fields in swamps, lotus fields, and rice fields	[55]
	Other agricultural land	Cultivation of wheat, upland rice, vegetables, grassland, lawns, apples, pears, peaches, grapes, tea, paulownia, water chestnuts, mulberry, and other perennial plants	
	Forests	Densely growing perennial plants	
	Wastelands	Wasteland, cliffs, rocks, perennial snow, wetlands, mining areas, etc.	
	Building sites	Densely built-up residential and urban areas	
	Roads	Roads and other areas with a flat surface	
	Railways	Railway lines, marshalling yards, etc.	
	Other sites	Sports grounds, airports, racecourses, baseball grounds, schools, port areas, open spaces in artificial land, etc.	
	Rivers and lakes	Artificial lakes, natural lakes, ponds, fish farms, etc. that are always full of water at low tide, and riverbeds in rivers and river areas	
	Seashores	Sand, gravel, and rocks that border the coast	
	Seawater areas	Rocky outcrops, mudflats, sea pans	
	Golf courses	Fairways and roughs of golf courses, and the border between forests and golf courses	

## Result and discussion

### Basic analysis

Table 5 presents the breakdown by municipality and disaster hazard of the percentage of young females at high hazard. The data indicates that the highest percentage for all disaster hazards is less than 10%. It means that in over 80% of municipalities, the percentage of young females at high hazard is relatively low.

**Table 5:** Percentage of young females at high-hazard in 2050.

Percentage of young females at high hazard	Flood		Tsunami		Storm-surge		Sediment	
91–100%	0	(0%)	0	(0%)	1	(0%)	0	(0%)
81–90%	1	(0%)	1	(0%)	1	(0%)	0	(0%)
71–80%	1	(0%)	2	(0%)	0	(0%)	0	(0%)
61–70%	3	(0%)	4	(1%)	0	(0%)	0	(0%)
51–60%	8	(1%)	2	(0%)	0	(0%)	0	(0%)
41–50%	8	(1%)	12	(2%)	2	(0%)	1	(0%)
31–40%	14	(2%)	13	(2%)	3	(0%)	0	(0%)
21–30%	39	(5%)	25	(3%)	2	(0%)	8	(1%)
11–20%	86	(12%)	40	(5%)	1	(0%)	34	(5%)
0%–10%	584	(78%)	645	(87%)	734	(99%)	701	(94%)

Focusing on the percentage of young females at high hazard by type of hazards, 35 and 34 municipalities have a percentage of young females at high hazard of flooding or tsunamis exceeding 30%. In contrast, only seven municipalities have over 30% of young females at high hazard storm surge, and just one municipality has a similar percentage for sediment-related disasters.

Tables 6–9 present the municipalities with the highest percentage of young females at high hazard for various hazards. These tables show the top 50 municipalities for floods and tsunamis, where the percentage of young females at high hazard is significant, while the top 20 for other hazards. The figures in brackets in the Municipality column indicate the names of the prefectures. The numbers for young females at high-hazard and low-hazard and the total citizen count represent projected population figures for 2050. The results show that flood (Table 6), tsunami (Table 7), and storm surge hazards (Table 8), the data includes both small municipalities with populations of a few hundred and medium-sized municipalities with populations numbering in the tens of thousands. Conversely, all top municipalities are small for sediment-related hazards (Table 9), with populations ranging from a few hundred to a few thousand. This difference arises because many municipalities listed above may face serious sediment-related hazards in mountainous areas.

**Table 6:** List of top municipalities by percentage of young females at high risk of flooding.

Rank	Municipality	Percentage of young females at high hazard	Number of young females at high hazard	Number of young females at low hazard	Number of citizens
1	Kawajima-machi (Saitama)	85%	445	80	11,022
2	Itakura-machi (Gunma)	77%	484	146	8,712
3	Kaizu-shi (Gifu)	68%	594	276	17,756
4	Yoshinogawa-shi (Tokushima)	68%	906	423	22,633
5	Yoshimi-machi (Saitama)	62%	257	159	9,671
6	Ochi-cho (Kochi)	57%	62	47	2,521
7	Arida-shi (Wakayama)	56%	484	379	14,597
8	Yoro-cho (Gifu)	56%	497	393	14,417
9	Kasagi-cho (Kyoto)	56%	5	4	367
10	Goka-machi (Ibaraki)	54%	128	111	4,534
11	Toyokoro-cho (Hokkaido)	53%	50	44	1,737
12	Shimanto-shi (Kochi)	53%	765	690	20,436
13	Shibetsu-shi (Hokkaido)	51%	175	166	8,012
14	Ino-cho (Kochi)	50%	202	204	10,940
15	Ozu-shi (Ehime)	50%	598	604	21,366
16	Tadami-machi (Fukushima)	48%	54	58	2,084
17	Hidaka-mura (Kochi)	48%	41	45	2,654
18	Iiyama-shi (Nagano)	48%	194	214	10,400
19	Kamiita-cho (Tokushima)	46%	198	230	6,884
20	Ikeda-cho (Hokkaido)	43%	32	42	3,100
21	Takahashi-shi (Okayama)	42%	382	528	14,031
22	Fuchu-shi (Hiroshima)	38%	602	980	20,800
23	Hiraizumi-cho (Iwate)	38%	99	163	3,790
24	Tsushima-shi (Aichi)	38%	1,045	1,728	42,290
25	Kuji-shi (Iwate)	36%	459	809	17,896
26	Katsuragi-cho (Wakayama)	35%	223	421	8,534
27	Okura-mura (Yamagata)	34%	17	33	1,346
28	Ibara-shi (Okayama)	33%	532	1,099	23,584
29	Kuma-mura (Kumamoto)	32%	25	52	650
30	Tamamura-machi (Gunma)	32%	507	1,067	25,742
31	Sagara-mura (Kumamoto)	32%	19	40	1,887
32	Sakuho-machi (Nagano)	32%	86	182	5,687
33	Bifuka-cho (Hokkaido)	32%	20	43	2,096
34	Marumori-machi (Miyagi)	31%	57	128	4,974
35	Kotake-machi (Fukuoka)	30%	45	103	3,742
36	Nakatombetsu-cho (Hokkaido)	29%	5	12	804
37	Niimi-shi (Okayama)	29%	158	386	14,693
38	Kamaishi-shi (Iwate)	29%	305	750	16,363
39	Nakayama-machi (Yamagata)	29%	99	245	6,417
40	Kaminokuni-cho (Hokkaido)	29%	14	35	1,639
41	Kawachi-machi (Ibaraki)	29%	36	90	3,897
42	Ando-cho (Nara)	28%	62	158	4,494
43	Fujikawa-cho (Yamanashi)	28%	127	325	8,617
44	Nishiizu-cho (Shizuoka)	28%	18	47	2,869
45	Nanbu-cho (Aomori)	27%	99	265	8,464
46	Higashimiyoshi-cho (Tokushima)	27%	167	454	8,184
47	Kakuda-shi (Miyagi)	27%	226	616	16,575
48	Kyowa-cho (Hokkaido)	26%	42	118	3,332
49	Setana-cho (Hokkaido)	26%	23	67	3,033
50	Imakane-cho (Hokkaido)	25%	40	117	2,513

**Table 7:** List of top municipalities by percentage of young females at high risk of tsunami.

Rank	Municipality	Percentage of young females at high hazard	Number of young females at high hazard	Number of young females at low hazard	Number of citizens
1	Kisosaki-cho (Mie)	82%	133	30	3,561
2	Shiranuka-cho (Hokkaido)	79%	63	17	2,841
3	Shikabe-cho (Hokkaido)	77%	112	33	1,778
4	Noboribetsu-shi (Hokkaido)	68%	1,143	545	26,963
5	Nahari-cho (Kochi)	63%	37	22	1,723
6	Samani-cho (Hokkaido)	62%	43	26	1,706
7	Hokuto-shi (Hokkaido)	60%	1,104	724	27,360
8	Kikonai-cho (Hokkaido)	57%	16	12	1,295
9	Nakatosa-cho (Kochi)	54%	63	54	2,494
10	Yakumo-cho (Hokkaido)	50%	174	177	8,382
11	Toyo-cho (Kochi)	47%	9	10	870
12	Minamiise-cho (Mie)	45%	25	30	3,427
13	Hamanaka-cho (Hokkaido)	45%	85	105	3,162
14	Sukumo-shi (Kochi)	45%	235	293	9,651
15	Tosashimizu-shi (Kochi)	44%	77	97	5,124
16	Nachikatsuura-cho (Wakayama)	44%	152	193	6,910
17	Aki-shi (Kochi)	43%	289	385	8,409
18	Komatsushima-shi (Tokushima)	43%	616	831	20,786
19	Onjuku-machi (Chiba)	42%	59	82	4,381
20	Akkeshi-cho (Hokkaido)	40%	89	131	4,343
21	Erimo-cho (Hokkaido)	40%	38	56	2,219
22	Kuroshio-cho (Kochi)	39%	44	69	4,971
23	Susaki-shi (Kochi)	38%	218	353	10,434
24	Urakawa-cho (Hokkaido)	38%	100	165	6,515
25	Tano-cho (Kochi)	38%	26	43	1,257
26	Kuji-shi (Iwate)	37%	473	795	17,896
27	Mugi-cho (Tokushima)	36%	13	23	1,382
28	Hachinohe-shi (Aomori)	35%	3,247	6,052	151,087
29	Kihoku-cho (Mie)	33%	86	174	6,336
30	Hirogawa-cho (Wakayama)	33%	62	128	3,941
31	Owase-shi (Mie)	31%	71	156	7,125
32	Kujukuri-machi (Chiba)	31%	93	210	7,210
33	Noda-mura (Iwate)	31%	23	52	2,104
34	Fudai-mura (Iwate)	30%	10	23	1,057
35	Shimamaki-mura (Hokkaido)	30%	6	14	649
36	Shiraoi-cho (Hokkaido)	30%	57	133	7,706
37	Oshamambe-cho (Hokkaido)	30%	42	100	2,454
38	Nishiizu-cho (Shizuoka)	29%	19	46	2,869
39	Kushiro-shi (Hokkaido)	29%	2,046	5,062	98,544
40	Happou-cho (Akita)	26%	10	28	2,831
41	Susami-cho (Wakayama)	26%	10	28	1,620
42	Suttsu-cho (Hokkaido)	26%	16	46	1,538
43	Minamiboso-shi (Chiba)	25%	173	506	19,201
44	Otsuchi-cho (Iwate)	24%	64	198	5,394
45	Mukawa-cho (Hokkaido)	24%	27	85	3,555
46	Mihama-cho (Wakayama)	24%	64	203	4,051
47	Kushimoto-cho (Wakayama)	24%	86	273	7,188
48	Ikata-cho (Ehime)	23%	21	69	3,502
49	Minamisanriku-cho (Miyagi)	23%	50	168	5,095
50	Minami-cho (Tokushima)	23%	38	128	2,633

**Table 8:** List of top municipalities by percentage of young females at high risk of storm-surge.

Rank	Municipality	Percentage of young females at high hazard	Number of young females at high hazard	Number of young females at low hazard	Number of citizens
1	Kisosaki-cho (Mie)	98%	160	3	3,561
2	Shiroishi-cho (Saga)	88%	644	85	12,558
3	Tsushima-shi (Aichi)	49%	1,363	1,410	42,290
4	Hirao-cho (Yamaguchi)	40%	195	290	6,667
5	Takehara-shi (Hiroshima)	36%	197	355	11,636
6	Hikawa-cho (Kumamoto)	31%	113	247	6,499
7	Omachi-cho (Saga)	31%	78	175	3,577
8	Etajima-shi (Hiroshima)	27%	123	331	10,232
9	Kamiamakusa-shi (Kumamoto)	21%	93	357	11,669
10	Chikujo-machi (Fukuoka)	17%	133	650	9,865
11	Amakusa-shi (Kumamoto)	7%	159	2,009	39,327
12	Taku-shi (Saga)	7%	37	521	10,306
13	Ashikita-machi (Kumamoto)	6%	21	305	6,880
14	Tabuse-cho (Yamaguchi)	6%	30	500	8,918
15	Genkai-cho (Saga)	5%	9	163	3,332
16	Minamata-shi (Kumamoto)	3%	26	962	12,700
17	Reihoku-machi (Kumamoto)	2%	3	130	3,537
18	Minamichita-cho (Aichi)	2%	9	398	7,839
19	Naruto-shi (Tokushima)	2%	49	2,447	33,701
20	Suooshima-cho (Yamaguchi)	1%	2	224	6,363

**Table 9:** List of top municipalities by percentage of young females at high risk of sediment.

Rank	Municipality	Percentage of young females at high hazard	Number of young females at high hazard	Number of young females at low hazard	Number of citizens
1	Yamato-son (Kagoshima)	45%	10	12	730
2	Okuwa-mura (Nagano)	26%	26	74	1,747
3	Hinohara-mura (Tokyo)	25%	6	18	1,037
4	Okutama-machi (Tokyo)	24%	12	39	2,659
5	Yoshino-cho (Nara)	21%	9	34	1,952
6	Niyodogawa-cho (Kochi)	21%	10	38	1,821
7	Hinoemata-mura (Fukushima)	21%	5	19	279
8	Nammoku-mura (Gunma)	20%	1	4	406
9	Sanagouchi-son (Tokushima)	20%	9	36	877
10	Mishima-mura (Kagoshima)	19%	3	13	219
11	Doshi-mura (Yamanashi)	18%	7	33	875
12	Higashiyoshino-mura (Nara)	17%	1	5	441
13	Kudoyama-cho (Wakayama)	16%	6	31	1,487
14	Toyo-cho (Kochi)	16%	3	16	870
15	Soni-mura (Nara)	16%	3	16	378
16	Owase-shi (Mie)	15%	35	192	7,125
17	Toyone-mura (Aichi)	15%	2	11	486
18	Yura-cho (Wakayama)	14%	10	59	2,567
19	Owani-machi (Aomori)	14%	14	83	3,642
20	Kanna-machi (Gunma)	14%	1	6	498

**Fig 9:**
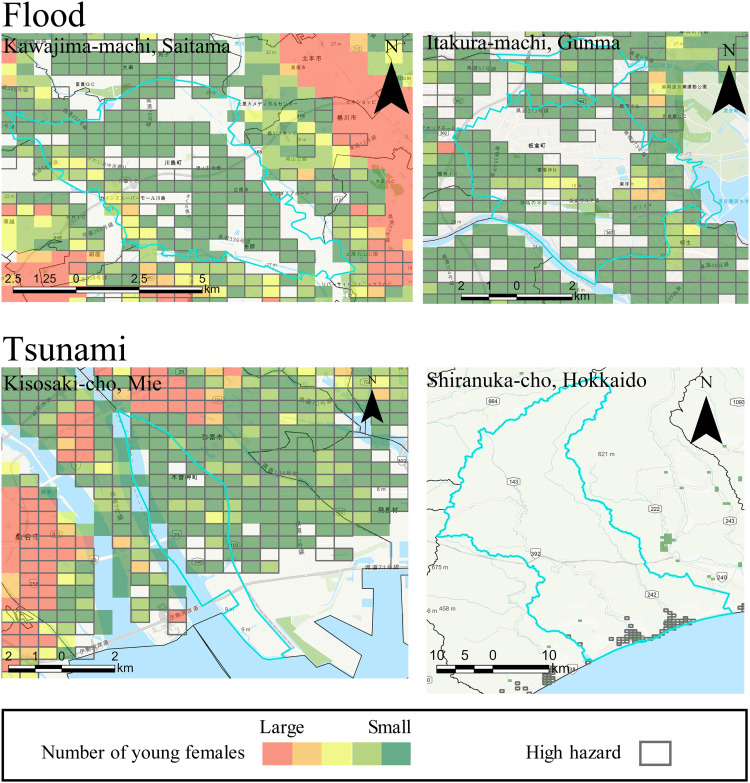
Examples of municipalities where a large proportion of the female population live within a high-hazard area.

**Fig 10:**
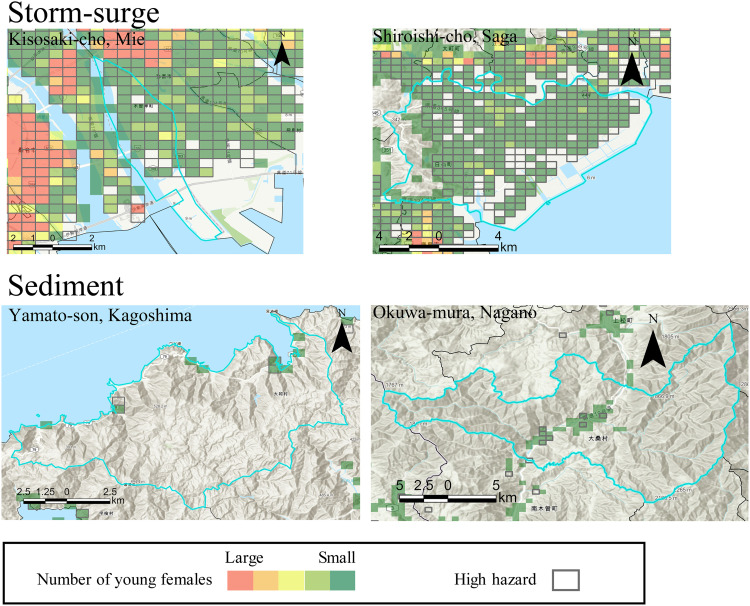
Examples of municipalities where a large proportion of the female population live within a high-hazard area (continues).

Figs 9 and 10 illustrate the distribution of the top two municipalities for each hazard. The leading municipalities for flood hazard are Kawajima-machi in Saitama Prefecture and Itakura-machi in Gunma Prefecture. Kawajima-machi is adjacent to the Arakawa River, a Class A river, and faces the risk of flooding if the embankment fails. Class A rivers are significant to the national economy and people’s lives, and the minister manages them [60,61]. Itakura-machi borders both the Tonegawa River and Watarasegawa River, also a Class A river, and similarly risks flooding if its embankments were to collapse. The top two municipalities for tsunami hazards are Kisosaki-cho in Mie Prefecture and Shiranuka-cho in Hokkaido. Kisosaki-cho, an urban area, is at risk of flooding from a tsunami. In Shiranuka-cho, most young females reside in a small coastal region susceptible to tsunami damage. The leading municipalities for storm surge hazards are Kisosaki-cho in Mie Prefecture and Shiroishi-cho in Saga Prefecture. Kisosaki-cho ranks first for tsunami hazards along the Kisogawa River, a Class A river, and thus faces risks of levee failure. Shiroishi-cho is vulnerable to damage from storm surges associated with typhoons, potentially affecting the entire municipality. The top two locations for sediment-related disaster hazards are Yamato-son in Kagoshima Prefecture and Okuwa-mura in Nagano Prefecture. Yamato-son is a small village projected to have a total population of 730 in 2050, with young females primarily concentrated in areas with steep slopes at risk for sediment-related disasters. Okuwa-mura, another small village in a mountainous region, is expected to have a population of 1,747 in 2050. A main road traverses the area and is at risk of sediment-related disasters.

### Spatial regression model

Tables 10–13 present the results of the spatial autoregressive models for different hazards. The dependent variable for all models is the percentage of young females at high hazard. We estimated these models by selecting independent variables to maximize the Adjusted R-squared while considering the Variance Inflation Factor (VIF). A VIF value above ten is often considered an indication of multicollinearity [62], and all models used in this study maintained VIF values of 10 or less. In the ordinary least squares (OLS) analysis of all models, the results of Moran’s I test rejected the hypothesis that the model residuals were independent and identically distributed. This finding indicates the presence of autocorrelation within the models.

**Table 10:** Results of the OLS estimations (Flood).

Variables		OLS	SAR	SEM	SDEM
Population		0.000 (0.000)	0.000 (0.000)	0.000 (0.000)	0.000 (0.000)
Ratio of elderly population		−0.099 (0.088)	−0.135 (0.083)	−0.157 (0.089)	−0.199 (0.089)*
Ratio of workers	primary sector	−0.05 (0.041)	−0.064 (0.039)	−0.07 (0.042)	−0.071 (0.042)
	secondary sector				
	tertiary sector				
Financial strength index					
Ratio of sewage treatment coverage		−0.036 (0.025)	−0.029 (0.024)	−0.023 (0.026)	−0.023 (0.027)
Number of public facilities					
Ratio of inhabitable area to total municipal area		0.064 (0.037)	0.066 (0.035)	0.081 (0.038)*	0.095 (0.04)*
Ratio of the area of each land use to the area of the municipality	Rice fields				
	Other agricultural land	−0.057 (0.064)	−0.047 (0.061)	−0.067 (0.066)	−0.066 (0.07)
	Forests				
	Wastelands				
	Building sites	0.174 (0.1)	0.173 (0.095)	0.115 (0.1)	0.132 (0.101)
	Roads				
	Railways	−3.834 (2.33)	−3.211 (2.215)	−2.867 (2.232)	−3.14 (2.231)
	Other sites	−0.82 (0.319)*	−0.714 (0.304)*	−0.821 (0.306)**	−0.829 (0.305)**
	Rivers and lakes	0.587 (0.126)**	0.542 (0.119)**	0.632 (0.128)**	0.638 (0.13)**
	Seashores	−3.135 (1.32)*	−2.711 (1.256)*	−2.521 (1.285)*	−2.51 (1.279)*
	Seawater areas	−0.054 (0.022)*	−0.039 (0.021)	−0.047 (0.022)*	−0.038 (0.022)
	Golf courses	−1.591 (0.591)**	−1.46 (0.562)*	−1.323 (0.615)*	−1.174 (0.646)
Constant		0.136 (0.049)**	0.127 (0.046)**	0.143 (0.05)**	0.144 (0.05)**
**Spatial effects**					
Spatial lag			0.465 (0.066)**		
Spatial error				0.526 (0.069)**	
Spatial lag and error					0.478 (0.073)**
**Model diagnostics**					
Wald test of spatial term			50.13**	58.29**	70.78**
Adjusted R-squared		0.087	0.089	0.099	0.136
AIC		−1145.201	−1187.500	−1191.861	−1188.712
Moran I		50.57**			
Standard error in parenthesis; **p < 0.01; *p < 0.05.					

**Table 11:** Results of the OLS estimations (Tsunami).

Variables		OLS	SAR	SEM	SDEM
Population		0.000 (0.000)	0.000 (0.000)	0.000 (0.000)	0.000 (0.000)
Ratio of elderly population		−0.084 (0.083)	−0.051 (0.077)	−0.063 (0.082)	−0.009 (0.083)
Ratio of workers	primary sector	0.155 (0.055)**	0.09 (0.052)	0.081 (0.06)	0.026 (0.061)
	secondary sector				
	tertiary sector	0.178 (0.065)**	0.122 (0.061)*	0.113 (0.069)	0.064 (0.07)
Financial strength index					
Ratio of sewage treatment coverage		−0.113 (0.023)**	−0.085 (0.022)**	−0.09 (0.024)**	−0.085 (0.024)**
Number of public facilities					
Ratio of inhabitable area to total municipal area		−0.101 (0.034)**	−0.068 (0.032)*	−0.071 (0.035)*	−0.038 (0.036)
Ratio of the area of each land use to the area of the municipality	Rice fields				
	Other agricultural land	−0.022 (0.059)	−0.017 (0.055)	−0.027 (0.06)	−0.047 (0.063)
	Forests				
	Wastelands	0.136 (0.16)	0.095 (0.149)	0.028 (0.16)	−0.059 (0.162)
	Building sites	−0.199 (0.093)*	−0.205 (0.086)*	−0.227 (0.091)*	−0.254 (0.092)**
	Roads				
	Railways	3.512 (2.148)	2.969 (2.001)	2.731 (2.033)	2.702 (2.033)
	Other sites	1.871 (0.288)**	1.848 (0.268)**	1.816 (0.273)**	1.755 (0.272)**
	Rivers and lakes	0.073 (0.115)	0.044 (0.107)	0.043 (0.116)	0.037 (0.118)
	Seashores	8.358 (1.199)**	7.817 (1.118)**	7.934 (1.154)**	7.781 (1.146)**
	Seawater areas				
	Golf courses	−1.072 (0.539)*	−0.896 (0.503)	−0.939 (0.557)	−0.573 (0.582)
Constant		0.036 (0.057)	0.027 (0.053)	0.059 (0.061)	0.061 (0.061)
**Spatial effects**					
Spatial lag			0.533 (0.060)**		
Spatial error				0.561 (0.064)**	
Spatial lag and error					0.512 (0.068)**
**Model diagnostics**					
Wald test of spatial term			78.68**	75.98**	88.48**
Adjusted R-squared		0.166	0.188	0.177	0.215
AIC		−1265.861	−1330.892	−1325.752	−1322.712
Moran I		70.10**			

Standard error in parenthesis; **p < 0.01; *p < 0.05.

**Table 12:** Results of the OLS estimations (Storm-surge).

Variables		OLS	SAR	SEM	SDEM
Population		0.000 (0.000)	0.000 (0.000)	0.000 (0.000)	0.000 (0.000)
Ratio of elderly population		0.049 (0.043)	0.048 (0.042)	0.061 (0.044)	0.063 (0.044)
Ratio of workers	primary sector				
	secondary sector				
	tertiary sector	0.02 (0.023)	0.017 (0.023)	0.015 (0.024)	0.01 (0.024)
Financial strength index					
Ratio of sewage treatment coverage		−0.012 (0.013)	−0.01 (0.012)	−0.008 (0.013)	−0.006 (0.014)
Number of public facilities					
Ratio of inhabitable area to total municipal area		0.003 (0.021)	0 (0.02)	0 (0.021)	−0.004 (0.023)
Ratio of the area of each land use to the area of the municipality	Rice fields	0.101 (0.029)**	0.1 (0.028)**	0.12 (0.03)**	0.139 (0.032)**
	Other agricultural land				
	Forests				
	Wastelands				
	Building sites	−0.061 (0.048)	−0.058 (0.047)	−0.066 (0.049)	−0.068 (0.051)
	Roads				
	Railways	−2.356 (1.158)*	−2.383 (1.129)*	−2.495 (1.134)*	−2.551 (1.141)*
	Other sites	1.253 (0.156)**	1.26 (0.153)**	1.271 (0.153)**	1.234 (0.154)**
	Rivers and lakes	0.005 (0.063)	0.008 (0.061)	0.021 (0.065)	0.042 (0.067)
	Seashores	−1.464 (0.644)*	−1.432 (0.628)*	−1.47 (0.639)*	−1.558 (0.643)*
	Seawater areas				
	Golf courses	−0.897 (0.287)**	−0.876 (0.28)**	−0.877 (0.303)**	−0.795 (0.33)*
Constant		−0.03 (0.026)	−0.029 (0.025)	−0.035 (0.027)	−0.033 (0.028)
**Spatial effects**					
Spatial lag			0.368 (0.094)**		
Spatial error				0.449 (0.088)**	
Spatial lag and error					0.383 (0.101)**
**Model diagnostics**					
Wald test of spatial term			15.25**	25.75**	30.23**
Adjusted R-squared		0.133	0.134	0.146	0.164
AIC		2188.451	−2200.078	−2208.140	−2195.344
Moran I		13.97**			

Standard error in parenthesis; **p < 0.01; *p < 0.05.

**Table 13:** Results of the OLS estimations (Sediment).

Variables		OLS	SAR	SEM	SDEM
Population					
Ratio of elderly population		0.193 (0.032)**	0.176 (0.031)**	0.175 (0.033)**	0.16 (0.032)**
Ratio of workers	primary sector				
	secondary sector				
	tertiary sector				
Financial strength index		0.018 (0.01)	0.016 (0.01)	0.015 (0.01)	0.011 (0.01)
Ratio of sewage treatment coverage		−0.002 (0.009)	0.001 (0.009)	0.002 (0.009)	0.007 (0.01)
Number of public facilities					
Ratio of inhabitable area to total municipal area		−0.057 (0.013)**	−0.046 (0.012)**	−0.045 (0.013)**	−0.033 (0.014)*
Ratio of the area of each land use to the area of the municipality	Rice fields				
	Other agricultural land	0.021 (0.021)	0.02 (0.02)	0.014 (0.022)	0.018 (0.024)
	Forests				
	Wastelands	−0.038 (0.06)	−0.021 (0.058)	−0.017 (0.061)	0.027 (0.065)
	Building sites	0.085 (0.037)*	0.069 (0.036)	0.053 (0.038)	0.018 (0.038)
	Roads	−0.418 (0.573)	−0.364 (0.557)	−0.308 (0.563)	−0.268 (0.558)
	Railways	−0.352 (0.817)	−0.117 (0.797)	−0.022 (0.804)	0.082 (0.796)
	Other sites	−0.046 (0.113)	−0.033 (0.11)	−0.035 (0.111)	−0.034 (0.11)
	Rivers and lakes	−0.009 (0.044)	−0.011 (0.043)	−0.017 (0.045)	−0.01 (0.047)
	Seashores	−0.464 (0.47)	−0.373 (0.458)	−0.333 (0.466)	−0.234 (0.465)
	Seawater areas	0.021 (0.008)*	0.023 (0.008)**	0.019 (0.008)*	0.013 (0.008)
	Golf courses	0.028 (0.211)	0.026 (0.206)	−0.032 (0.22)	−0.16 (0.241)
Constant		−0.047 (0.018)*	−0.048 (0.018)**	−0.042 (0.019)*	−0.038 (0.019)*
**Spatial effects**					
Spatial lag			0.295 (0.066)**		
Spatial error				0.357 (0.075)**	
Spatial lag and error					0.188 (0.084)**
**Model diagnostics**					
Wald test of spatial term			19.73**	22.93**	48.52**
Adjusted R-squared		0.137	0.152	0.152	0.206
AIC		−2706.248	−2721.005	−2722.972	−2727.202
Moran I		21.16**			

Standard error in parenthesis; **p < 0.01; *p < 0.05.

A common characteristic observed across all the models is that the adjusted R-squared values are low. A general guideline for assessing model fit is an adjusted R-squared of 0.5 or higher [63]. However, in this study, most models had adjusted R-squared values ranging from approximately 0.1 to 0.2, which is significantly below the recommended threshold of 0.5. The likely reasons for this low performance include external factors that are not accounted for by the independent variables selected in this study. Specifically, there may be other variables beyond municipal land use, socioeconomic factors, and infrastructure conditions that affect the dependent variable. Additionally, there might be inherent limitations in modeling these factors effectively. Therefore, this study focuses on the significant independent variables from the models that showed a relatively better fit, keeping in mind the limitations previously discussed, and proceed with the analysis. The following discussion focuses on each hazard individually.

For flooding (Table 10), the model with the highest Adjusted R-squared is the SDEM model, which has a value of 0.136. This model reveals that the percentage of young females at high hazards is positively and significantly related to the “Ratio of inhabitable areas to total municipal area,” “Rivers and lakes.” Conversely, it is negatively and significantly related to “Ratio of elderly population,” “Other sites,” and “Seashores.” In other words, municipalities with a high percentage of livable areas, low aging rates, and many rivers and lakes in inland areas have a higher percentage of young females exposed to flooding. It supports hypothesis 1, which suggests that the percentage increases when residential areas and rivers spread across the entire municipal area. It is also related to hypothesis 5, which states that low aging rates are one of the social conditions.

**Fig 11:**
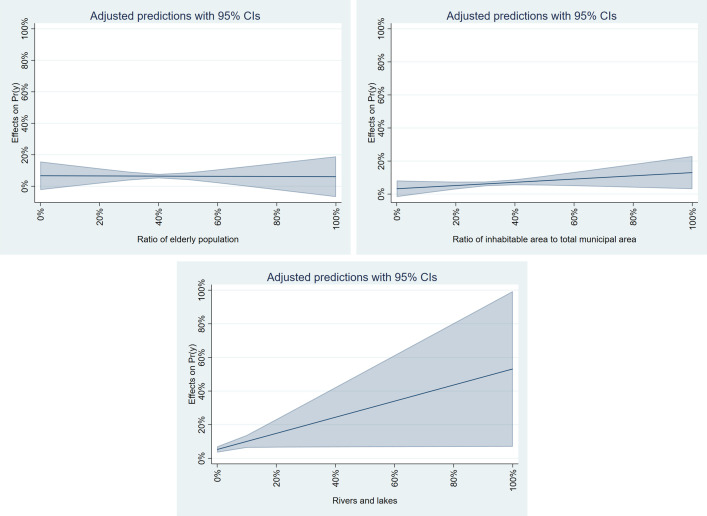
Marginal effects of SDEM model for flooding.

To further analyze the results of the SDEM model, we calculated the marginal effects. Fig 11 illustrates the marginal effects for three significant independent variables, which resulted in a positive estimated value of a dependent variable using the Stata command “margins.” [64]. Since the marginal effects of the independent variables “Other sites” and “Seashores” were negative, we excluded these variables from the analysis. This figure shows how the estimated value of the dependent variable (the percentage of young females at high risk) changes when the target independent variable varies and other independent variables are held constant at their mean values. The shaded areas in the figures represent the 95% confidence interval. The figure indicates that the most significant impact among the three variables is “Rivers and lakes.” It shows that an increase in the proportion of rivers—a geographical characteristic—leads to a significant rise in young females at high risk.

In the results for tsunamis (Table 11), the highest Adjusted R-squared for the SDEM model is 0.215. It indicates that the percentage of young females at high hazard is positively and significantly related to “Other sites” and “Seashores” while being negatively and significantly related to “Ratio of sewage treatment coverage” and “Building sites.” Therefore, in coastal municipalities with low sewerage coverage and few buildings, the percentage of young females exposed to tsunamis is high. It supports hypothesis 2, which suggests that the percentage is high in small suburban communities. It also supports hypothesis 5, suggesting that infrastructure development lags in such areas.

**Fig 12:**
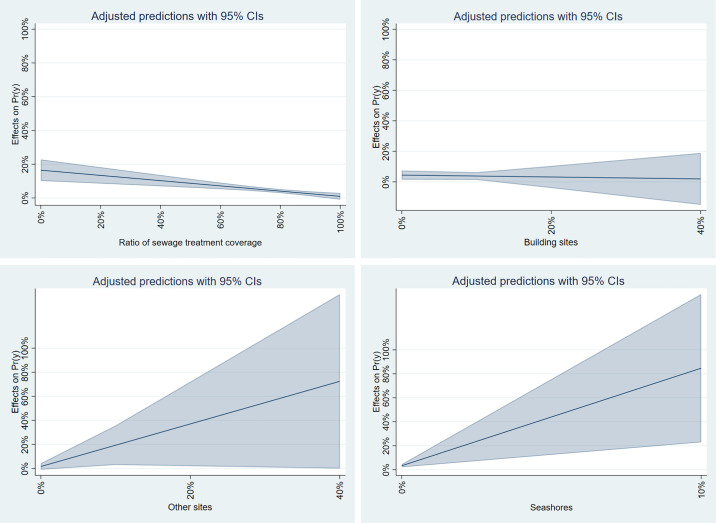
Marginal effects of SDEM model for tsunami.

Fig 12 presents the results of the marginal effects calculation for significant variables in the SDEM model for flooding. For “Other sites” and “Seashores,” the marginal effect significantly increased with a higher proportion, whereas a decrease was observed for “Building sites.” It suggests that young females are at risk of tsunami exposure, especially in municipalities with many lands except buildings facing the coast.

For storm surge results (Table 12), the model with the highest Adjusted R-squared is also the SDEM model, with a value of 0.164. This model shows that the percentage of young females at high hazards is positively and significantly related to “Rice fields” and “Other sites” while negatively and significantly related to “Railways,” “Seashores,” and “Golf courses.” It implies that municipalities with many rice fields and fewer beaches are more likely to experience storm surges. It supports hypothesis 3, indicating a high ratio in regions where rice paddies are prevalent in low-lying areas. Conversely, hypothesis 5, which relates to social, economic, and infrastructure factors, was not supported.

The proportion of hazard information for storm surges is significantly lower than for floods and tsunamis. As of 2020, the availability of flood hazard maps and tsunami hazard maps was 98% and 91%, respectively, while only 12% of storm surge hazard maps had been prepared [43]. In Kisosaki-cho and Shiroishi-cho, which exhibit the highest percentage of young females at high hazard, the proportion of coastal areas is 0%. In the southern part of Kisosaki-cho, there is a large amusement park, and in Shiroishi-cho, there are significant reclaimed areas and tidal flats.

**Fig 13:**
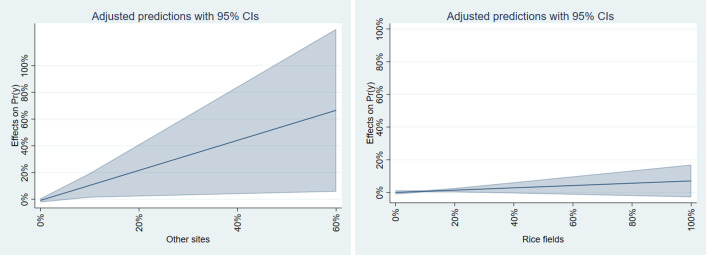
Marginal effects of SDEM model for storm-surge.

Fig 13 displays the results of calculating the marginal effects of significant variables in the SDEM model for storm surge. The variables “Railways,” “Seawater areas,” and “Golf courses” were excluded from the analysis because their results were either greater than or equal to 1 or contained negative values. The figure highlights that the variability in “Other sites” is particularly high, suggesting that a larger proportion of young females face storm surges in suburban areas. In contrast, the marginal effects of “Rice fields” are relatively low, indicating that the previously mentioned phenomena may only occur in specific rice fields.

Regarding sediment-related disasters (Table 13), the model with the highest Adjusted R-squared is the SDEM model, with a value of 0.206. This model indicates that the “Ratio of the elderly population” is positively and significantly associated with the percentage of young females at high hazard. In contrast, “Ratio of inhabitable area to total municipal area” is negatively and significantly related. It expresses that municipalities along the coast with smaller, more inhabitable areas have a higher percentage of young females exposed to sediment-related disasters. This fact reflects the mountainous and rural areas and does not apply to the newly developed residential areas assumed in Hypothesis 4. On the other hand, it supports Hypothesis 5 regarding the progression of aging in mountainous and rural areas.

**Fig 14:**
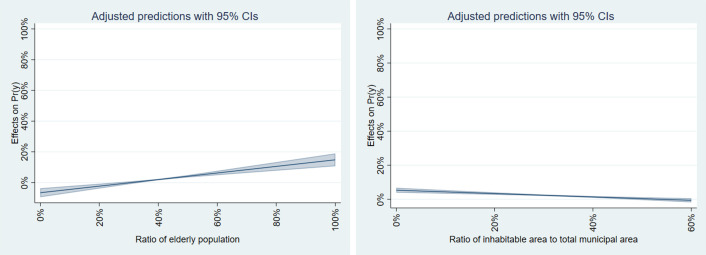
Marginal effects of SDEM model for sediment.

Fig 14 presents the results of calculating the marginal effects of significant variables in the SDEM model related to sediment. It shows that an increase in the aging rate has a greater impact on the proportion of young females exposed to sediment-related disasters than a decrease in habitable areas (Fig 14). This finding underscores the disaster risk concerns prevalent in mountainous and rural areas with a high proportion of elderly residents.

## Discussion

Many municipalities at risk of vanishing are small local governments with small populations. Implementing appropriate land use measures for young females living in these areas may mitigate disaster damage to homes and prevent population decline. This study aims to identify local governments where a high proportion of young females are exposed to various hazards and to analyze trends in land use statistically, and propose countermeasures.

The parameter estimation results for flooding indicate that young females exposed to flooding correlate with a significant proportion of ratio of elderly population, habitable land, inland locations, and areas abundant in rivers and lakes. The data from Kawajima-machi (Saitama) and Itakura-machi (Gunma) ranked first and second in exposed populations support the findings from the model.

In Japan, between 1960 and the 2000s, urbanization resulted in the development of suburban housing estates and the expansion of urban areas [65]. During the 2000s, the government established information on flood inundation scenarios and created legal frameworks for flood prevention [66]. However, once urban areas have expanded, it is challenging to reduce their size, even in the face of potential water-related disasters.

The government enacted the new law named “River Basin Disaster Resilience and Sustainability by All” principle in 2021 to deal with the increase in flood disaster risks due to global warming. It is promoting initiatives to prevent and reduce flooding, limit the number of affected individuals, and mitigate damage [67]. Regarding land use measures, it is challenging to relocate residents to safer areas in municipalities where habitable land at high risk of flooding is expanding in the municipality. Therefore, it is essential to enhance flood countermeasures, such as implementing building regulations in high-hazard residential areas, to reduce disaster-related damage to homes.

People have a strong preference for homeownership, and the decision to buy a home often aligns with significant life events, such as marriage and childbirth [68]. Once individuals acquire a home, they typically remain there for over 30 years [69]. A parameter estimation model indicates that the proportion of young females living in areas prone to flooding is higher in municipalities with lower aging rates. Therefore, it is essential to implement measures that prevent young females from choosing residential areas at high risk for flooding disasters.

Results for tsunami-related hazards reveal that the percentage of young females exposed to tsunamis is higher in municipalities facing the coast with fewer buildings and low sewage treatment coverage. The significant difference in findings compared to flooding is due to the insignificant impact of habitable land areas within these municipalities. It implies that areas with older settlements lack adequate infrastructure.

In coastal areas, the aging population is increasing due to a decline in the number of people working in the fishing industry [70]. The results from the model’s parameter estimation partially support the relationship between low sewerage coverage and a high percentage of the population being at risk. The challenge is how to protect residents of small communities from tsunamis.

In Japan, pre-disaster recovery planning, informed by the Great East Japan Earthquake lessons, is being promoted nationwide [71,72]. For municipalities where conventional residential areas are vulnerable to tsunami damage, it is crucial to provide tsunami-safe housing by expanding habitable land through projects such as relocation to higher ground. However, if the project is implemented under the current framework in Japan, it is important to note that the transfer cost may exceed the disaster prevention cost [73].

The parameter estimation for storm surges indicates that young females exposed to these events correlate in areas with shared characteristics such as “rice fields” and “other sites.” At the same time, “railways,” “seashores,” and “golf courses” are scarce. Similar to floods, storm surges can lead to flooding damage. The result reveals that it frequently impacts homes in suburban areas with prevalent rice fields and farmland. Given the challenges of implementing cost-effective hardware improvements for these residences, implementing housing regulations will mitigate damage to storm surges.

Finally, the estimation results for sediment-related disasters show that young females correlate with a limited amount of habitable area, many “seawater areas,” and areas with a low aging ratio. It is evident in Yamato-son (Kagoshima) and Okuwa-mura (Nagano), which ranked first and second in exposed populations. These houses are in narrow coastal or mountainous habitable zones, often in small municipalities with minimal populations.

The municipalities identified in this study as being at risk of sediment share similarities with those exposed to tsunamis. Specifically, they are older villages with a significant proportion of elderly residents and have recently experienced a rapid population decline [74]. Therefore, it is feasible to support the relocation of buildings to reduce sediment-related disasters rather than implementing large-scale hardware measures [46].

## Conclusion

This paper presented the first comprehensive risk analysis of the population in all potentially endangered municipalities in Japan. It calculated the potential exposure of the future young female population to four types of disaster hazards—floods, tsunamis, storm surges, and sediment-related disasters—at the municipal level, using precise data at a 500-meter mesh scale. Using a spatial regression model, we integrated land use data to visualize the factors affecting the exposed population. The relationship between the hypotheses in this study and the results is as follows.

(1) The model validated hypothesis 1 regarding flooding. It demonstrated that municipalities with larger inhabitable areas and more rivers existing in the city tend to have a higher proportion of residents exposed to flooding.
(2) The model also validated hypothesis 2 concerning tsunamis. It showed that municipalities located in sparsely built coastal suburban areas tend to have a higher proportion of residents exposed to tsunamis.
(3) The model partially validated hypothesis 3 regarding storm surge. Due to data limitations, this study was unable to include elevation as a variable, which prevented the accurate representation of low-lying environments. However, by using rice fields as an alternative variable for low-lying areas, the model revealed that municipalities with a higher proportion of rice fields have a higher proportion of residents exposed to storm surge.
(4) The model did not confirm hypothesis 4 concerning sediment. The hypothesis suggested that municipalities where the population increased due to new housing developments in mountainous areas during Japan’s period of rapid economic growth (around 1960–1970) would have higher sediment. However, the model indicated that municipalities with villages established before the period, or those with a small population, tend to have a higher proportion of residents exposed to sediment.
(5) Hypothesis 5, which discusses the impact of social, economic, and infrastructure development, was partially validated by some models. The flood model indicated that municipalities with a lower proportion of elderly population tend to have a higher proportion of residents exposed to flooding. The tsunami model revealed that municipalities with lower sewage treatment coverage tend to have a higher proportion of residents who are at risk of being exposed to tsunamis. Additionally, the sediment model indicated that municipalities with smaller inhabitable areas and a higher proportion of elderly population tend to have a higher proportion of residents exposed to sediment.

The analysis clarified the relationship between various land types—such as habitable land, farmland, buildings, rivers, inland areas, and coastal areas—and the young female population exposed to different hazards at the national level. For instance, in the context of flooding, it was discovered that some municipalities lacked residential areas where inhabitants could avoid disasters. In the case of tsunamis, the analysis identified the potential for relocation to higher ground, influenced by the amount of habitable land available in each municipality. Furthermore, it revealed that building regulations should be enforced for suburban homes due to the risks associated with storm surges and that houses should be relocated from high-risk areas in the case of sediment-related disasters.

The approach utilized in this study demonstrates that individual municipalities cannot address disaster risk measures for municipalities in Japan facing potential disappearance alone. In areas with no safe living locations other than those susceptible to disasters, options for disaster prevention are severely limited. This finding raises new concerns in the current situation, where many municipalities compete to attract young residents.

## Supporting information

S1 File Average marginal effects estimated from the SDEM model for each disaster. (DOCX)
