# Supplementary material for: Spatial factors affecting young females’ disaster exposure in municipalities facing population decline
Source: PLoS One. 2025 Oct 17;20(10):e0334706. doi: 10.1371/journal.pone.0334706 (PMC12533900; doi:10.1371/journal.pone.0334706)
Supplement: S1 File — (DOCX) [file pone.0334706.s001.docx]

Table 10-a Average marginal effects of SDEM estimations (Flood)

| Variables | | dy/dx | std. err. | z | P>z | 95% conf. | interval |
| --- | --- | --- | --- | --- | --- | --- | --- |
| Population | |  |  |  |  |  |  |
| Ratio of elderly population | | -0.006 | 0.111 | -0.050 | 0.957 | -0.223 | 0.211 |
| Ratio of workers | primary sector | -0.038 | 0.077 | -0.500 | 0.619 | -0.190 | 0.113 |
|  | secondary sector |  |  |  |  |  |  |
|  | tertiary sector |  |  |  |  |  |  |
| Financial strength index | |  |  |  |  |  |  |
| Ratio of sewage treatment coverage | | -0.088 | 0.042 | -2.080 | 0.037 | -0.170 | -0.005 |
| Number of public facilities | |  |  |  |  |  |  |
| Ratio of inhabitable area to total municipal area | | 0.000 | 0.000 | -1.600 | 0.110 | 0.000 | 0.000 |
| Ratio of the area of each land use to the area of the municipality | Rice fields |  |  |  |  |  |  |
|  | Other agricultural land | -0.024 | 0.122 | -0.200 | 0.845 | -0.264 | 0.216 |
|  | Forests |  |  |  |  |  |  |
|  | Wastelands |  |  |  |  |  |  |
|  | Building sites | 0.404 | 0.265 | 1.530 | 0.127 | -0.115 | 0.923 |
|  | Roads |  |  |  |  |  |  |
|  | Railways | -9.819 | 6.164 | -1.590 | 0.111 | -21.899 | 2.261 |
|  | Other sites | -1.584 | 1.014 | -1.560 | 0.118 | -3.572 | 0.404 |
|  | Rivers and lakes | 0.479 | 0.242 | 1.980 | 0.048 | 0.005 | 0.954 |
|  | Seashores | -8.046 | 3.520 | -2.290 | 0.022 | -14.946 | -1.147 |
|  | Seawater areas | -0.040 | 0.090 | -0.440 | 0.659 | -0.217 | 0.137 |
|  | Golf courses | -2.116 | 1.211 | -1.750 | 0.081 | -4.490 | 0.258 |

Table 11-a Average marginal effects of SDEM estimations (Tsunami)

| Variables | | dy/dx | std. err. | z | P>z | 95% conf. | interval |
| --- | --- | --- | --- | --- | --- | --- | --- |
| Population | | 0.000 | 0.000 | 1.890 | 0.059 | 0.000 | 0.000 |
| Ratio of elderly population | | -0.048 | 0.149 | -0.320 | 0.749 | -0.340 | 0.245 |
| Ratio of workers | primary sector | 0.200 | 0.093 | 2.160 | 0.031 | 0.018 | 0.381 |
|  | secondary sector |  |  |  |  |  |  |
|  | tertiary sector | 0.135 | 0.109 | 1.240 | 0.214 | -0.078 | 0.349 |
| Financial strength index | |  |  |  |  |  |  |
| Ratio of sewage treatment coverage | | -0.155 | 0.040 | -3.880 | 0.000 | -0.233 | -0.077 |
| Number of public facilities | |  |  |  |  |  |  |
| Ratio of inhabitable area to total municipal area | | -0.187 | 0.070 | -2.670 | 0.008 | -0.325 | -0.050 |
| Ratio of the area of each land use to the area of the municipality | Rice fields |  |  |  |  |  |  |
|  | Other agricultural land | -0.007 | 0.118 | -0.060 | 0.954 | -0.238 | 0.224 |
|  | Forests |  |  |  |  |  |  |
|  | Wastelands | 0.789 | 0.352 | 2.240 | 0.025 | 0.098 | 1.480 |
|  | Building sites | -0.063 | 0.251 | -0.250 | 0.803 | -0.554 | 0.429 |
|  | Roads |  |  |  |  |  |  |
|  | Railways | 8.324 | 5.671 | 1.470 | 0.142 | -2.790 | 19.438 |
|  | Other sites | 1.770 | 0.955 | 1.850 | 0.064 | -0.101 | 3.641 |
|  | Rivers and lakes | 0.150 | 0.226 | 0.660 | 0.507 | -0.293 | 0.593 |
|  | Seashores | 8.137 | 3.181 | 2.560 | 0.011 | 1.902 | 14.371 |
|  | Seawater areas |  |  |  |  |  |  |
|  | Golf courses | -0.912 | 1.108 | -0.820 | 0.411 | -3.084 | 1.260 |

Table 12-a Average marginal effects of SDEM estimations (Storm surge)

| Variables | | dy/dx | std. err. | z | P>z | 95% conf. | interval |
| --- | --- | --- | --- | --- | --- | --- | --- |
| Population | | 0.000 | 0.000 | -0.860 | 0.387 | 0.000 | 0.000 |
| Ratio of elderly population | | 0.065 | 0.063 | 1.040 | 0.299 | -0.058 | 0.188 |
| Ratio of workers | primary sector |  |  |  |  |  |  |
|  | secondary sector |  |  |  |  |  |  |
|  | tertiary sector | 0.033 | 0.043 | 0.780 | 0.435 | -0.050 | 0.116 |
| Financial strength index | |  |  |  |  |  |  |
| Ratio of sewage treatment coverage | | -0.025 | 0.020 | -1.230 | 0.218 | -0.064 | 0.015 |
| Number of public facilities | |  |  |  |  |  |  |
| Ratio of inhabitable area to total municipal area | | 0.027 | 0.042 | 0.650 | 0.515 | -0.054 | 0.108 |
| Ratio of the area of each land use to the area of the municipality | Rice fields | 0.070 | 0.056 | 1.260 | 0.209 | -0.040 | 0.180 |
|  | Other agricultural land |  |  |  |  |  |  |
|  | Forests |  |  |  |  |  |  |
|  | Wastelands |  |  |  |  |  |  |
|  | Building sites | -0.147 | 0.124 | -1.190 | 0.236 | -0.389 | 0.096 |
|  | Roads | 0.385 | 3.031 | 0.130 | 0.899 | -5.556 | 6.326 |
|  | Railways |  |  |  |  |  |  |
|  | Other sites | 1.124 | 0.529 | 2.130 | 0.034 | 0.088 | 2.161 |
|  | Rivers and lakes | -0.028 | 0.120 | -0.240 | 0.813 | -0.264 | 0.207 |
|  | Seashores | -1.816 | 1.648 | -1.100 | 0.270 | -5.046 | 1.414 |
|  | Seawater areas |  |  |  |  |  |  |
|  | Golf courses | -0.911 | 0.578 | -1.580 | 0.115 | -2.045 | 0.222 |

Table 13-a Average marginal effects of SDEM estimations (Sediment)

| Variables | | dy/dx | std. err. | z | P>z | 95% conf. | interval |
| --- | --- | --- | --- | --- | --- | --- | --- |
| Population | |  |  |  |  |  |  |
| Ratio of elderly population | | 0.214 | 0.036 | 6.010 | 0.000 | 0.144 | 0.284 |
| Ratio of workers | primary sector |  |  |  |  |  |  |
|  | secondary sector |  |  |  |  |  |  |
|  | tertiary sector |  |  |  |  |  |  |
| Financial strength index | | 0.020 | 0.018 | 1.120 | 0.261 | -0.015 | 0.055 |
| Ratio of sewage treatment coverage | | -0.011 | 0.013 | -0.800 | 0.424 | -0.037 | 0.016 |
| Number of public facilities | |  |  |  |  |  |  |
| Ratio of inhabitable area to total municipal area | | -0.099 | 0.021 | -4.690 | 0.000 | -0.140 | -0.058 |
| Ratio of the area of each land use to the area of the municipality | Rice fields |  |  |  |  |  |  |
|  | Other agricultural land | 0.048 | 0.034 | 1.410 | 0.158 | -0.019 | 0.114 |
|  | Forests |  |  |  |  |  |  |
|  | Wastelands | -0.153 | 0.113 | -1.360 | 0.175 | -0.373 | 0.068 |
|  | Building sites | 0.260 | 0.089 | 2.940 | 0.003 | 0.087 | 0.434 |
|  | Roads | -1.240 | 1.384 | -0.900 | 0.370 | -3.953 | 1.473 |
|  | Railways | -2.382 | 1.995 | -1.190 | 0.232 | -6.292 | 1.527 |
|  | Other sites | -0.415 | 0.357 | -1.160 | 0.245 | -1.114 | 0.285 |
|  | Rivers and lakes | 0.043 | 0.074 | 0.590 | 0.556 | -0.101 | 0.187 |
|  | Seashores | -1.741 | 1.146 | -1.520 | 0.129 | -3.987 | 0.505 |
|  | Seawater areas | 0.014 | 0.030 | 0.470 | 0.640 | -0.045 | 0.073 |
|  | Golf courses | 0.102 | 0.389 | 0.260 | 0.794 | -0.661 | 0.865 |
